# Pore-Level Study of Syngas Production From Fuel-Rich Partial Oxidation in a Simplified Two-Layer Burner

**DOI:** 10.3389/fchem.2019.00793

**Published:** 2019-11-29

**Authors:** Junrui Shi, Mingming Mao, Houping Li, Yongqi Liu, Yasong Sun

**Affiliations:** ^1^School of Transportation and Vehicle Engineering, Shandong University of Technology, Zibo, China; ^2^Shaanxi Key Laboratory of Thermal Sciences in Aero-engine System, School of Power and Energy, Northwestern Polytechnical University, Xi'an, China

**Keywords:** pore level, fuel-rich partial oxidation, porous media, syngas production, staggered arrangement

## Abstract

We performed pore-level simulation of fuel-rich partial oxidation of a CO_2_/CH_4_ mixture in a two-dimensional porous burner with staggered arrangement of discrete particles. The chemistry was treated with detailed chemical kinetics GRI-Mech 1.2, and surface-to-surface radiation was taken into account by the discrete ordinates (DO) model. The predicted results were validated against the available experimental data and results by the volume-averaged method. The predicted main syngas products (CO, H_2_, and CO_2_) agreed well with the experimental data for the whole investigation range; it indicated that the pore-level simulation could precisely predict syngas productions from fuel-rich partial oxidation in a two-layer burner with the simplified arrangement of porous media. Variations of species, temperature, and velocity within the pores were presented and discussed. The predicted molar fractions of CO, H_2_, CO_2_, H_2_O, etc. over the pores between particles were highly two-dimensional; the flame thickness was on the order of the particle diameter (2.5 mm) and smaller than the particle diameter. The predicted area-weighted average temperatures were greater than the experiments due to the ignorance of the heat loss to the surroundings through burner walls. The effect of CO_2_ adding on syngas production is examined.

## Highlights

- Prediction of syngas production in pore level with detailed chemical mechanism.
- Surface-to-surface radiation is considered.
- The predicted velocities, temperatures, and species are highly two-dimensional.
- The maximum local velocity in the pores is 17.4 times of the interstitial gas velocity.
- 2D pore level with simplified geometry captures the syngas components.

## Introduction

Porous media burners have been widely used for syngas production from fuel-rich partial oxidation in porous media (Kennedy et al., 1999). A review by Oliveira and Kaviany (2001) examined the non-equilibrium of transport processes for combustion in porous media. Mujeebu (2016) presented a detailed review of hydrogen and syngas production by superadiabatic combustion. He pointed out that modeling was still an open problem and the effect of porous structures on syngas production needs further investigation.

The concept of employing superadiabatic combustion for hydrogen production was first reported by Weinberg et al. (1988), who experimentally proved that fuel-rich combustion can be maintained in spouted beds. Two types of porous burner for syngas production can be found in the literature based on porous material or its structure. For the first type, burners filled with homogeneous media were used, in which the combustion wave propagated in the upstream or downstream direction of the flow, mainly depending on the equivalence ratio and mixture velocity (Drayton et al., 1998; Kennedy et al., 2000).

To confine the combustion wave within packed bed, Kennedy et al. (1999) proposed and developed reciprocal flow burner (RFB) for syngas production. Rich flammability limit was successfully extended to an equivalence ratio of 8 for methane/air mixture. Zheng et al. (2012) modeled RFB with external heat source using a one-dimensional two-temperature model with GRI-Mech 1.2. Gonzalez et al. (2018) conducted an experimental study on syngas production from biogas and polyethylene in a porous burner; it was shown that the conversion efficiency was increased by the presence of polyethylene. Futko (2003) analyzed the chemical structure of fuel-rich partial oxidation in a porous burner with the use of skeleton diagrams and sensitivity analysis, and they suggested that combustion waves could be divided into three regions based on heat release: a preheating zone, an exothermic zone characterized by partial oxidation of methane, and an endothermic zone with the conversion processes.

For the second approach, burners with different porous material or structures and two-layer porous burners were used to confine the flame near the interface of two sections under a certain range of equivalence ratio and gas velocity (Pedersen-Mjaanes et al., 2005). Zeng et al. (2017) presented an experimental and numerical study on the influence of adding CO_2_ on syngas production in a two-layer burner. In their experimental study, the conversion efficiency was found to be increased from 39.1 to 45.3% when the molar ratio of CO_2_ to CH_4_ was increased from 0 to 1. They conducted a numerical study applying a two-dimensional two-temperature model based on the volume-averaged treatment. In their experimental study (Wang et al., 2018), the burner was optimized based on conversion efficiency, which was determined to be composed of 2.5-mm pellets in the upstream section and 7.5-mm pellets in the downstream section.

Volume-averaged treatment (Liu et al., 2009; Li et al., 2015; Yang et al., 2018; Fan et al., 2019) and pore-level simulation on porous media combustion have been applied for last three decades with special attention on fuel-lean combustion. Much of early work was limited to simple geometry with symmetry or periodic boundary conditions due to computational limitations. Sahraoui and Kaviany (1994) presented simulations of premixed combustion in packed bed for fuel-lean methane/air mixture with one-step chemistry. The volume-averaged method and the pore-level model were applied to study the superadiabatic effect, flame structure, and flame speed. Their pore-level model revealed that the significant variation of temperature and species concentration occurred over pores. However, solid radiation was ignored in their computations to save computational cost. Hackert et al. (1999) extended the work of Sahraoui and Kaviany (1994) by consideration of solid phase radiation.

Pereira et al. (2010) numerically studied porous media diffuser reformer using quasi-1D and three-dimensional models. Fuel-rich partial oxidation in the porous media was simulated by quasi-1D model with 12-step reduced chemistry. Bedoya et al. (2015) presented a comprehensive study of premixed combustion in porous media with special attention on the effects of pressure and equivalence ratio on flame speed. Both the volume-averaged model and the pore-level method were employed for three different porous burners. The three-dimensional porous structure for simulations was reconstructed with the help of computed tomography from a real sample of porous media. Considerable variation of temperature along space coordinate was predicted by the pore-level model.

Shi et al. (2017) reported combustion characteristics of fuel-lean mixture in packed bed made of discrete particles. They found significant thermal non-equilibrium between the same phase and interphase. Recently, Jiang et al. (2017, 2018) modeled filtration combustion in a random packed bed filled with pellets by large eddy model, and the turbulent-flame interaction in porous media with special porous structure was quantitatively analyzed.

As reviewed above, numerical studies on syngas production based on volume-averaged treatment have been extensively studied with different chemical kinetics for several fuels and rather development has been made. However, volume-averaged treatments filter out the detailed information at the smaller scales (Oliveira and Kaviany, 2001). Meanwhile, measurements of velocities, species profiles, temperature distributions, and flame structures were often difficult due to the obstacle of porous media. The knowledge on detailed information in pore level was scarce. To the authors' best knowledge, no pore-level numerical study on the syngas production was reported.

The detailed local simulations helped to understand the physics of filtration combustion in pore level. Faced with the complex three-dimensional geometry structures and high computational cost, pore-level simulation for simplified geometry structures with detailed kinetics was feasible at present and might present some principles for the future three-dimensional pore-level simulation with complex structures of porous media.

The aim of this work was to investigate syngas production from partial oxidation of fuel-rich CO_2_/CH_4_ mixture in a two-layer burner made of two-dimensional simplified geometries. The chemistry was treated with detailed chemical kinetics Gri-Mech 1.2 and surface-to-surface radiation between the pellets was taken into account using the discrete ordinates (DO) model.

## Physical Model

**Figure 1:**
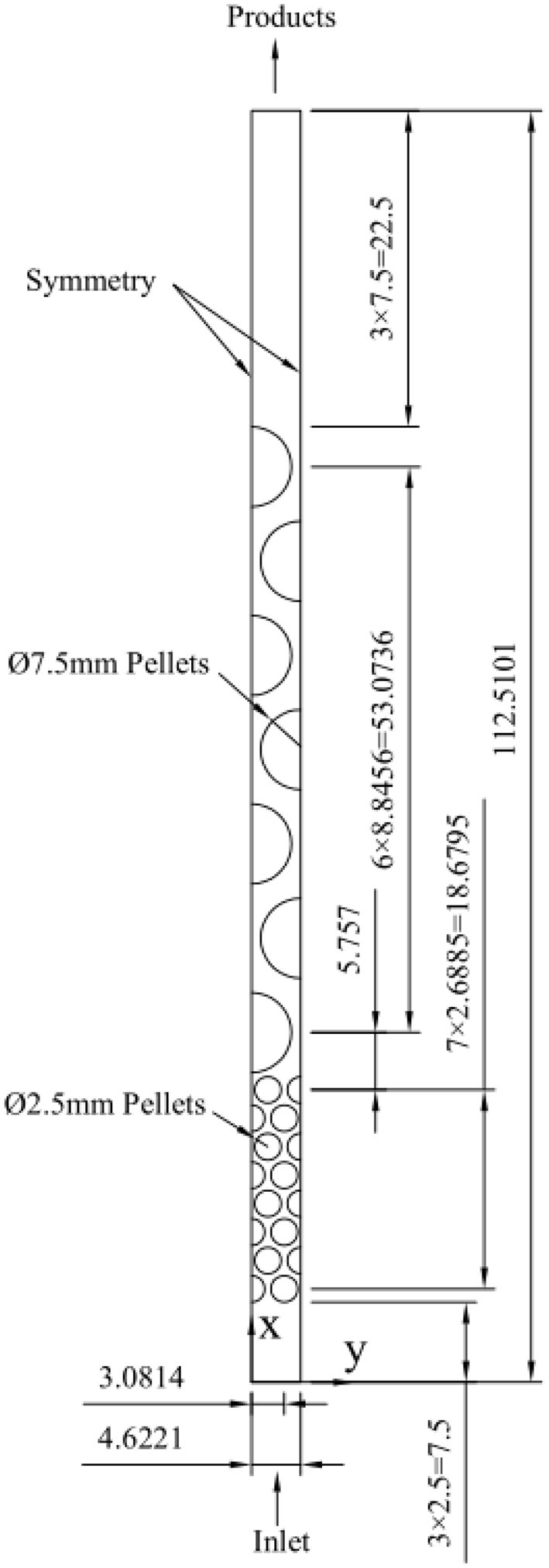
Schematic of the two-layer burner.

A two-layer burner developed and tested by Zeng et al. (2017) is considered in computations. The porous burner was composed of 2- to 3-mm alumina pellets with a length of 20 mm in the upstream and 7.5-mm alumina pellets in the downstream that was 60 mm long. The burner developed by Zeng et al. (2017) is a random packed bed, and three-dimensional pore-level simulations of syngas production in this random packed bed with detailed kinetics are infeasible due to computational limitations. Meanwhile, it is very difficult to use a 2D direct model for the random packed bed. Even for the 3D simple cubic and body center cubic packing, it is difficult to choose a 2D representative part to represent 3D geometry. In this study, the random packed bed is simplified to a two-dimensional structural arrangement of discrete cylinder particles. The process of reconstructing geometries is as follows. For a packed bed with 2.5-mm pellets in the upstream section and 7.5-mm pellets in the downstream section, we assume that all the particles are arranged in equilateral triangles and the corresponding porosities are computed as ε = 0.375+0.34*d*/*D*, where *d* and *D* are the diameter of the particle and burner, respectively. Under the above assumptions, the number of pellets and the distance between the neighboring pellets in the computational domain are determined. For simplification, just two rows of 2.5-mm particles and one row of 7.5-mm particles with symmetric boundary conditions at the right and left of the selected rows have been considered in the present study, as shown in Figure 1. The packed bed contains 16 cylindrical particles with a diameter of 2.5 mm in the upstream section and seven cylindrical particles with a diameter of 7.5 mm in the downstream section. The lengths of the corresponding zone filled with 2.5- and 7.5-mm particles are 21.1795 and 60.5736 mm, which are close to the lengths of the modeled burner (Zeng et al., 2017). It extends 7.5 mm in the upstream (inlet section) and 22.5 mm in the downstream (outlet section).

To simplify the problem, the following assumptions are made:

1. The pellets are opaque and inert homogeneous; solid surface scattering is ignored.
2. The surface-to-surface radiation and gas radiation are taken into account and computed by the DO model. The gas is gray gas and does not scatter; the refractive index of the gas is one.
3. The flow of gas mixture in the packed bed is assumed to be laminar.
4. Heat loss through burner walls to the surrounding is assumed to be proportional to the heat loss coefficient β and the difference between the local and ambient temperature.

Under the above assumptions, a set of differential equations can be obtained.

Continuity equation

(1) $$\begin{array}{r} \nabla \cdot \left(\rho_{g}\text{v}\right)=0 \end{array}$$

where ρ_*g*_ represents the gas density and **v**denotes the velocity vector.

Momentum equation

(2) $$\begin{array}{r} \nabla \cdot \left(\rho_{g}\text{v}v_{i}\right)=\nabla \cdot \left(\mu \nabla v_{i}\right)-\frac{\partial P}{\partial x_{i}} \end{array}$$

where *v*_*i*_ represents the horizontal or vertical velocity, μ is dynamic viscosity, and *P* is the pressure.

Gas phase energy equation

(3) $$\begin{array}{l} \nabla \cdot \left(\rho_{g}c_{g}\text{v}T_{g}\right)=\nabla \cdot \left(\lambda_{g}\nabla T_{g}\right)+\underset{i}{\sum }\omega_{i}h_{i}W_{i}-\nabla \cdot q_{r}\\ \;-\beta \left(T_{g}-T_{0}\right) \end{array}$$

where *T*_g_, λ_g_, *c*_*g*_ are the gas temperature, thermal conductivity, and specific heat, respectively. ω_i_, *W*_*i*_ are the chemical reaction rate and molecular weight of species *i*, respectively. β is heat loss coefficient (Contarin et al., 2003). *q*_R_ is radiation flux, obtained by the DO model coupled to the model for the properties of gas mixture. The DO model solves radiative transfer equation (RTE) in the direction $\vec{s}$ as a field equation. For model gas radiation, we assume that the gas is gray gas and does not scatter, the refractive index of the gas is one, and the RTE for the gas phase in this study is simplified to:

$$\begin{array}{l} \nabla \cdot \left(I\left(\vec{r},\vec{s}\right)\vec{s}\right)+\alpha I\left(\vec{r},\vec{s}\right)=\alpha \sigma T^{4}\surd \pi \end{array}$$

where $I\left(\vec{r},\vec{s}\right)$ is radiation intensity, σ is Stefan–Boltzmann constant, and α is gas absorption coefficient and computed by weighted sum of gray gases model. The heat source term due to radiation that appears in Equation (3) is calculated from RTE: $\nabla \cdot q_{r}=4\alpha \sigma T^{4}-\alpha G$, where *G* is incident radiation.

Solid phase energy equation

(4) $$\begin{array}{r} \nabla \cdot \left(\lambda_{s}\nabla T_{s}\right)-\beta \left(T_{s}-T_{0}\right)=0 \end{array}$$

where *T*_*s*_, λ_*s*_ is the solid temperature and thermal conductivity, respectively.

Species conservation equation

(5) $$\begin{array}{r} \nabla \cdot \left(\rho_{g}\text{v}Y_{i}\right)-\nabla \cdot \left(\rho_{g}D_{i}\nabla Y_{i}\right)-\omega_{i}W_{i}=0 \end{array}$$

where *D*_i_, *Y*_*i*_ is diffusion coefficient and mass fraction of species *i*, respectively.

## Boundary Conditions

The following boundary conditions are specified in the model:

1. Inlet
  (6) $$\begin{array}{r} T_{g}=T_{s}=300K,u=u_{0},v=0\\ {\;Y}_{\mathrm{CH}4}={Y_{\mathrm{CH}4}}_{,in},Y_{O2}={Y_{O2}}_{,in},Y_{\mathrm{CO}2}={Y_{\mathrm{CO}2}}_{,in} \end{array}$$
2. Outlet
  (7) $$\begin{array}{r} \frac{\partial T_{g}}{\partial x}=\frac{\partial T_{s}}{\partial x}=\frac{\partial \left(Y_{i}\right)}{\partial x}=\frac{\partial \left(u\right)}{\partial x}=0 \end{array}$$
3. Radiative heat loss from burner inlet and outlet is considered;
  (8) $$\begin{array}{r} \lambda_{s}\frac{\partial T_{s}}{\partial x}=-\varepsilon_{r}\sigma \left({T^{4}}_{s,\text{in}/\text{out}}-{T_{0}}^{4}\right) \end{array}$$
  ε_*r*_ is the solid surface emissivity and σ is the Stefan–Boltzmann constant.
4. At *y* = 0, 3.0814 mm, symmetry conditions are imposed;
  (9) $$\begin{array}{r} \frac{\partial T_{g}}{\partial y}=\frac{\partial T_{s}}{\partial y}=\frac{\partial Y_{i}}{\partial y}=\frac{\partial u}{\partial y}=v=0 \end{array}$$

At the gas–solid interfaces, the non-slip condition is imposed for gas velocity. The symbol used in this work is summarized in Table 1. Table 2 presents the values of velocity, temperature, and pressure at the burner inlet.

**Table 1:** Symbols used in this work.

**Nomenclature**	
*d*	Diameter of spheres, m
*p*	Pressure, Pa
*T*_0_	Ambient temperature, K
*v*	Horizontal velocity, m/s
*x*	Vertical coordinate, m
*y*	Horizontal coordinate, m
*h_*i*_*	The molar enthalpy of species *i*, kJ/kg
*T*	Temperature, K
*u*	Vertical velocity, m/s
*W_*i*_*	Molecular weight of species *i*, kg/kmol
*X*	Molar fraction
*Y*	Mass fraction
**Greek symbols**	
ϕ	Equivalent ratio
ρ	Density, kg/m^3^
ω_i_	Reaction rate of species *i*, kmol/m^3^ s
α	Volume flow ratio between CO_2_ and CH_4_
η_e−s_	Syngas energy conversion efficiency
λ	Thermal conductivity, W/m·K
ε	Porosity
σ	Stephan-Boltzmann constant, W/m^2^·K^4^
μ	Dynamic viscosity, Pa·s
*ε_*r*_*	Solid surface emissivity
**Subscripts**	
β	Heat loss coefficient, W/m^3^ × K
g	Gas
s	Solid

**Table 2:** Numerical values at boundary inlet.

Pressure	1.013e^5^ Pa
The molar ratio of CO_2_/CH_4_ and its corresponding inlet velocity (m/s)	0 (0.1365), 0.25 (0.1412), 0.5 (0.1458), 1 (0.1551)
Gas temperature	300 K

## Initial Conditions, Material Porperties, and Solution

The governing Equations (1)–(5) with the boundary conditions are numerically solved by a CFD software Fluent 15.0. The chemistry is treated with detailed kinetics Gri-Mech 1.2; it includes 32 species and 177 reactions. The thermal and transport properties of gas are obtained from the Chemkin and Tranfit packages (Kee et al., 1986). These properties and detailed reaction mechanism are imported into Fluent 15.0. Detailed chemical mechanisms are invariably numerically stiff and compute-intensive. However, we used direct integration for the chemical reaction. Fluent 15.0 computes the Arrhenius rate for the laminar flow. The thermal conductivity of the alumina pellets is specified as polynomial functions of the temperature (Munro, 2010). The density of pellets is specified as 3,984 kg/m^3^. The absorption coefficient and emissivity of the pellets are set to be 80 m^−1^ and 0.4 (Henneke and Ellzey, 1999).

The pressure field is solved using the SIMPLE method. At the interface of the upstream and downstream sections, the solid temperature of pellets is set to be 2,200 K to model the ignition process. We have tested the sensitivity of this initial heating to the predicted results. It is shown that the ignition fails when the initial heating temperature is smaller than 2,200 K. A residual error of 10^−6^ for energy equations and 10^−3^ for all other equations are taken as convergence criteria.

The conversion efficiency is defined as (Zeng et al., 2017):

(10) $$\begin{array}{r} \eta_{e-s}=\frac{Y_{CO}\times LHV_{\mathrm{CO}}+Y_{H2}\times LHV_{H2}}{Y_{CH4,in}\times LHV_{CH4}} \end{array}$$

where LHV_CH4_, LHV_CO_, and LHV_H2_, are the low heating value of CH_4_, CO, and H_2_, respectively.

## Mesh Independence Study

**Figure 2:**
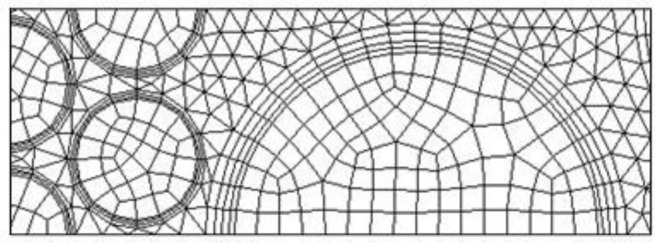
Mesh in the vicinity of the interface between the two sections.

As shown in Figure 1, the computational domain includes inlet section, outlet section, and solid and fluid zone. The fluid zone is constantly changing and the fluid path is tortuous. Chemical reaction takes place at the fluid zone, intense convective heat transfer occurs near the particle surfaces in the fluid side, and thus more mesh is expected in the fluid zone. In this study, non-uniform grid system is used in the computation, with finer grid in the fluid zone. Three mesh configurations are analyzed during the convergence study for three levels, coarse (mesh 1), average (mesh 2), and fine (mesh 3). Table 3 shows the details of the mesh size and mesh type in the computations. Figure 2 shows the mesh in the vicinity of the interface between the 2.5- and 7.5-mm pellets. Three prism layers are applied at the fluid side for all the particle surfaces. Quad mesh is used in the solid zone, and triangle mesh is used in the fluid zone.

**Table 3:** Mesh configurations.

	**Mesh 1**	**Mesh 2**	**Mesh 3**
Inelt part (quad mesh)	1	0.5	0.5
Solid zone (quad mesh)	1	0.5	0.25
Fluid zone (tri mesh)	1	0.5	0.25
Prism layers	0.05 × 1.2 × 3 (fisrt row × growth factor × prism layers) for 2.5-mm particles; 0.11 × 1.2 × 4 for 7.5-mm particles		
Outlet part (quad mesh)	1	0.5	0.5
Total mesh	2017	3876	4668

To estimate the influence of the mesh resolution, we analyze the average temperature and normalized velocity (divided by *u*_0_/ε) in the burner. The average temperature is defined as average value perpendicular to the flow direction. The heating of the cold particle (300 K) with hot air (1,600 K) is calculated, including the radiation, but the chemical reaction is not considered; other parameters and the solution procedure are the same to the full problem.

**Figure 3:**
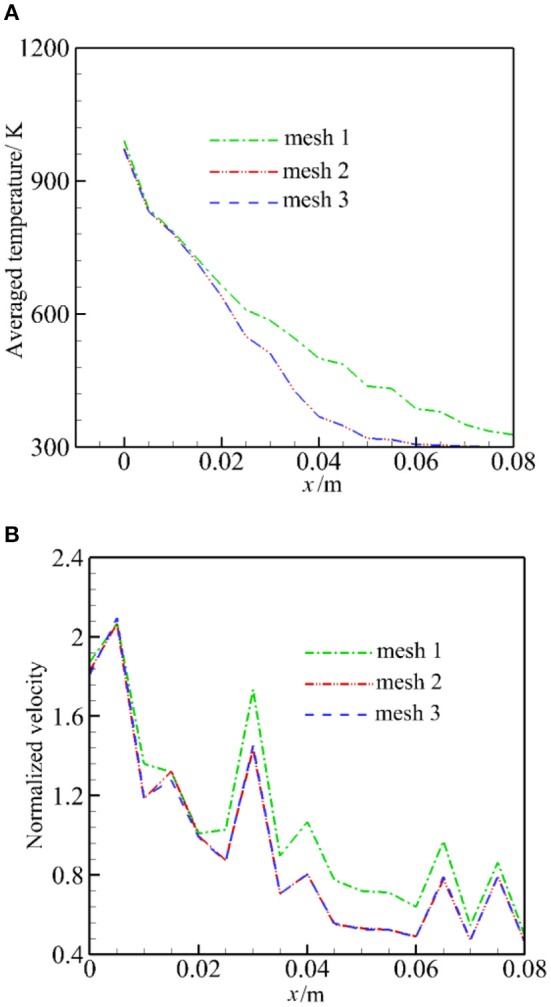
Averaged temperature and normalized velocity for three mesh configurations. **(A)** Averaged temperature. **(B)** Normalized velocity.

Figure 3 presents the predicted average temperature and normalized velocity for the three mesh configurations. It can be seen that the average temperature and normalized velocity for mesh 1 differs considerably from other configurations. It is shown that mesh resolution has a larger impact on flow parameter than the heat transfer. The predicted average temperatures by mesh 2 and mesh 3 are almost the same over the entire range. The predicted normalized velocity demonstrates the largest difference of 3.5% between mesh configurations 2 and 3. Finally, mesh 2 was used for the computation.

## Results and Discussion

### Species Profiles, Temperature Distributions, and Velocities Within Pores

In the experiment (Zeng et al., 2017), the equivalence ratio (φ) and air flow rate are set to be 1.5 and 5 L/min, while the ratio (α) between CO_2_ and CH_4_ is changed from 0 to 1. In the computation, for all computed cases, the equivalence ratio for methane/air mixture is set to be a fixed value of 1.5 and the mixture velocity at the burner inlet is varied due to the adding CO_2_ in fuel.

**Figure 4:**
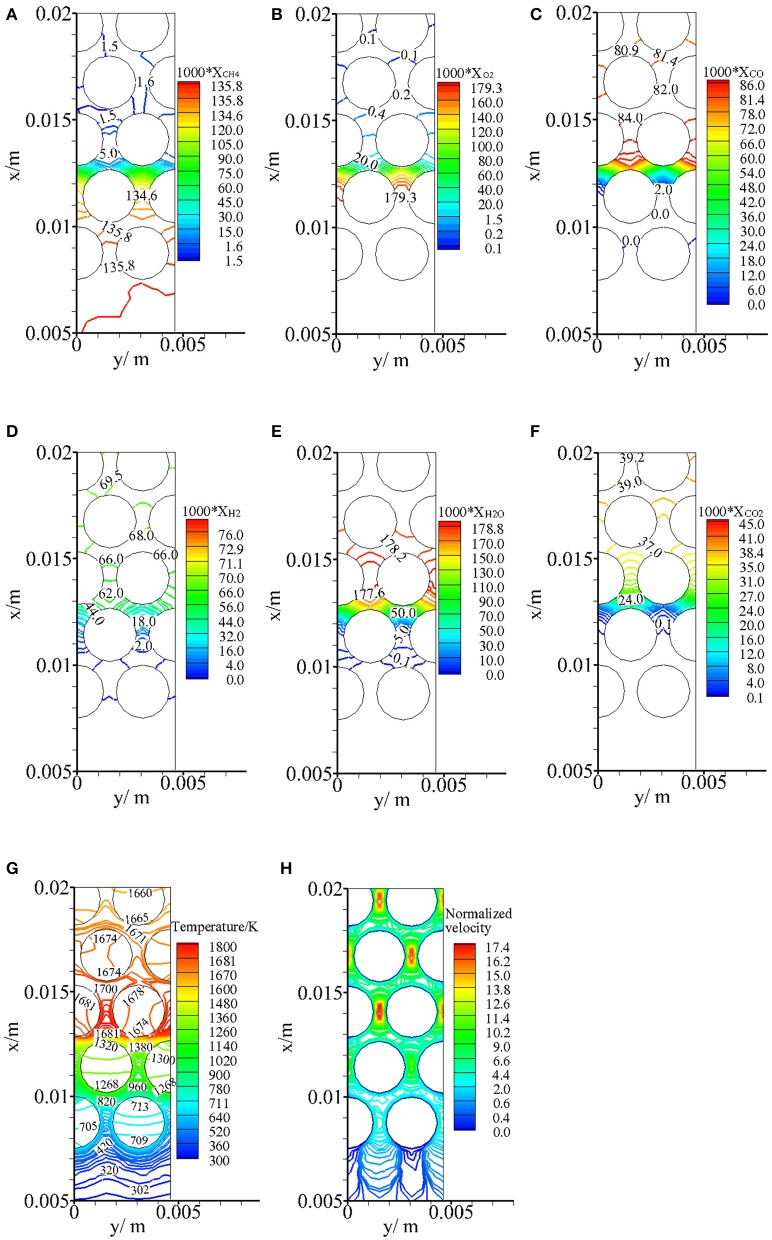
Predicted molar fractions of CH_4_, O_2_, CO, H_2_, H_2_O, CO_2_, temperature profiles, and normalized velocity distributions near the exothermic zone for α = 0 and ϕ = 1.5. **(A)** *X*_CH4_, **(B)** *X*_O2_, **(C)** *X*_CO_, **(D)** *X*_H2_, **(E)** *X*_H2O_, **(F)** *X*_CO2_, **(G)** Temperature (K), and **(H)** Normalized velocity.

Figure 4 illustrates the predicted molar fractions of CH_4_, O_2_, CO, H_2_, H_2_O, and CO_2_ (*X*_CH4_, *X*_O2_, *X*_CO_, *X*_H2_, *X*_CO2_, *X*_H2O_), temperature, and normalized velocity near the exothermic zone for α = 0 and φ = 1.5. One can see that the distributions of syngas components in the pores are highly two-dimensional. Slight decreases in *X*_CH4_ and *X*_O2_ are observed between the second and third rows of the particles. This indicates that CH_4_ begins to break down and a very smaller O_2_ is consumed in the preheating zone. Then, significant decreases in *X*_CH4_ and *X*_O2_ are observed within the pores between the second row of particles and it means that extensive chemical reaction occurs within a small region. The thickness of the exothermic zone is on the order of pellet diameter (2.5 mm) and smaller than the pellet diameter. Predictions (Zeng et al., 2017) by the volume-averaged method showed that the thickness of flame zone was smaller than 3 mm for this case. Our predictions of flame thickness agree with the results by the volume-averaged method. The main syngas components, CO and H_2_, are being formed over the pores between the second and third rows of particles, and then a slight reduction in *X*_CO_ and increase in *X*_H2_ are revealed in the post-flame zone. *X*_H2O_ peaks just after the exothermic zone and a slight decrease in *X*_H2O_ is observed in the post-flame zone, whereas *X*_CO2_ still slightly increases after the exothermic zone. The small variations of molar fraction of CO, CO_2_, H_2_, and H_2_O in the endothermic zone are captured by our model. For fuel-rich partial oxidation in porous media, the water–gas shift reaction CO+H2O → H2+CO2 is an important reaction in the reforming process, which leads to a slight increase in *X*_H2_ and *X*_CO2_ and a slight decrease in *X*_CO_ and *X*_H2O_.

The high gas temperature zone is located just downstream the reaction zone. In the post-flame zone, as shown in Figure 4G, the temperature difference within the same particle is rather small and can be negligible. This is because adequate heat exchange takes place between the gas and solid phases through convective heat transfer, and the solid thermal conductivity is greater compared to that of the gas phase. However, in the preheating region, obvious thermal non-equilibrium is observed within the same pellet. This is the result of reaction heat redistribution and multimode heat transfer mechanisms in the packed bed. The combustion takes place near the second row of the particles, and part of the reaction heat is stored in the porous media around the reaction zone through convective heat transfer between the gas and solid phases. Then, solid radiation occurs between the heated particles and the neighboring particles. The particles of the first row are heated via surface-to-surface radiation. Thus, heat recirculation is realized through solid radiation from the downstream high-temperature region to the upstream low-temperature region. As a result, the incoming fresh mixtures prior to entering the combustion zone are preheated by gas radiation and through convective heat transfer between the gas and solid phases. It is shown in Figure 4G that the gas mixture is substantially preheated before entering the reaction zone.

The normalized velocity is defined as the ratio of the predicted local velocity to the average interstitial gas velocity. As shown in Figure 4H, the maximum normalized velocity reaches up to 17.4 and this means that the maximum velocity in the pores is 17.4 times of the interstitial gas velocity. The distribution of normalized velocity is periodic by the distance of particle diameter and this is due to the staggered arrangement of the particles.

### Temperature Distributions

**Figure 5:**
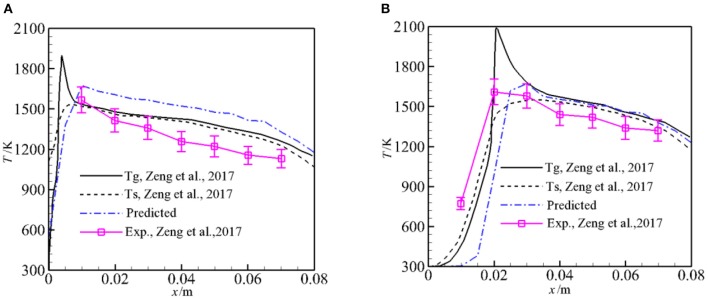
Predicted temperatures for ϕ = 1.5 and α = 0, 1. **(A)** Predicted temperatures for α = 0 and ϕ = 1.5. **(B)** Predicted temperatures for α = 1 and ϕ = 1.5.

Figure 5 presents the experimental values, the predicted solid temperatures by Zeng et al. (2017), and the predicted averaged temperatures along the flow direction for φ = 1.5 and α = 0, 1. It is noted that the experimental values and predictions (Zeng et al., 2017) in Figure 5 are the temperatures along the burner centerline. To compare the predicted temperatures by the pore-level model with the experiments and predictions, the predicted temperatures in this work in Figure 5 are area-weighted average temperatures in the line perpendicular to the flow direction. Our prediction shows the same trend as experiments, but the predicted averaged temperatures are greater than the experiments and the predictions (Zeng et al., 2017) especially for α = 0. The difference is mainly due to the model used in present study. In our model, radiation heat loss to the surrounding from the burner inlet and outlet is considered. However, only part of the burner in the flow direction is considered and symmetry conditions are used, heat loss to the surroundings through burner walls is taken into account by an empirical formula. Zeng et al. (2017) modeled the syngas production using the same physics model, in their two-dimensional model, heat loss to the surroundings through the burner walls was taken into account and good agreement between their predictions and experiments was observed compared to our model. For α = 1, both the predictions by Zeng et al. (2017) and this work show that the flame shifts slightly to downstream compared to the experimental results, and the predicted *T*_s_ and the average temperature agree well with the experiment when the measurement error is taken into account.

### Combustion Products and Conversion Efficiencies

**Figure 6:**
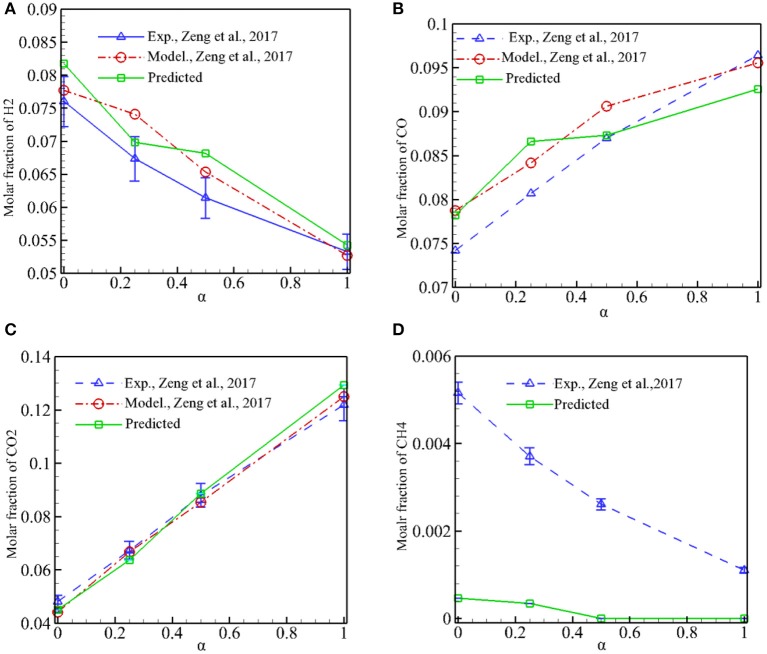
Predicted molar fraction of H_2_, CO, CO_2_, and CH_4_ in the exhaust gases as a function of α for ϕ = 1.5. **(A)** Predicted *X*_H2_ in the exhaust gases. **(B)** Predicted *X*_CO_ in the exhaust gases. **(C)** Predicted *X*_CO2_ in the exhaust gases. **(D)** Predicted *X*_CH4_ in the exhaust gases.

Figure 6 shows the predicted molar fraction of major combustion products (H_2_, CO, CO_2_, CH_4_) in the exhaust gases as a function of α for φ = 1.5, and experimental results and numerical predictions by Zeng et al. (2017) are also presented for comparison. It is shown in Figure 6 that *X*_CO_ and *X*_CO2_ increases, while *X*_H2_ and *X*_CH4_ reduces when α is increased from 0 to 1. It can be noted that our model can successfully capture the syngas components with different α. The predictions by our model and Zeng et al. (2017) show good agreement with the experimental results for the whole range of α. Our predictions of *X*_H2_ are slightly greater than the experimental values, and the differences between the predictions and experiments are reasonable when the measurement error is taken into account. The amount of unburned CH_4_ in the exhaust gases is very small and *X*_CH4_ is overpredicted by our model. It is noted that our model has almost the same accuracy in predicting the major combustion products as the volume-averaged method used by Zeng et al. (2017). This indicates that modeling syngas production in pore level with staggered arrangement of discrete particles is feasible and the predictions are reliable. Meanwhile, as shown in Figure 4, detailed information in the pores such as velocity profiles, temperature distributions, and syngas components can be obtained by the present model; this information is often difficult to measure in experiments, and it is very important to deeply understand the physics of syngas combustion in porous media.

**Figure 7:**
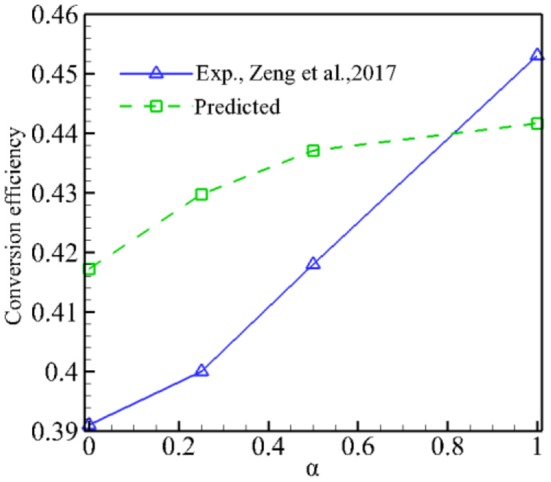
Predicted conversion efficiency as a function of α for ϕ = 1.5.

Figure 7 presents the predicted conversion efficiency of syngas production as well as experimental values as a function of α for φ = 1.5. Both the predictions and experiments show that the conversion efficiency increases with α; it indicates that adding CO_2_ in the fuel can increase the conversion efficiency. Our model predicts the same trend as experiments, but the conversion efficiency is overpredicted for 0 ≤ α ≤ 0.5 compared to the experimental results. Again, this is due to the fact that the model overpredicts the combustion temperature and therefore the conversion efficiency.

## Conlusions

A numerical study on syngas production from fuel-rich partial oxidation in a two-layer burner made of staggered arrangements of particles is conducted using detailed chemistry mechanism GRI-Mech 1.2. The predicted results for the temperature, syngas production of components, and conversion efficiency are compared with experiments and the results from volume-averaged method. The major conclusions are as follows:

1. The pore-level modeling of syngas production with the simplified discrete particles captures the feature of fuel-rich combustion in pore level. The model can successfully capture the syngas components with different molar ration of CO_2_/CH_4_.
2. The thermal non-equilibrium in the same particle is observed in the burner. The thermal non-equilibrium varies along the flow direction and is not obvious in the post-flame zone.
3. The detailed information of syngas components, temperature, and velocity over the pores is presented with the pore-level model, which is difficult to get in experiment.
4. The predicted molar fractions of CH_4_, CO, H_2_, and CO_2_ in the pores are highly two-dimensional. The maximum gas velocity in the pores is 17.4 times that of the interstitial gas velocity.

## Data Availability Statement

The datasets generated for this study are available on request to the corresponding author.

## Author Contributions

JS, MM, and HL conceived and designed the study. HL and YL analyzed the data. MM and YS wrote the manuscript.

### Conflict of Interest

The authors declare that the research was conducted in the absence of any commercial or financial relationships that could be construed as a potential conflict of interest.
